# Design and Stereochemical Research (DFT, ECD and Crystal Structure) of Novel Bedaquiline Analogs as Potent Antituberculosis Agents

**DOI:** 10.3390/molecules21070875

**Published:** 2016-07-04

**Authors:** Yiding Geng, Linwei Li, Chengjun Wu, Yumeng Chi, Zhen Li, Wei Xu, Tiemin Sun

**Affiliations:** 1 Key Laboratory of Structure-Based Drug Design and Discovery, Shenyang Pharmaceutical University, Ministry of Education, Shenyang 110016, China; gengyiding86@163.com (Y.G.); xt_llw@163.com (L.L.); chengjun871@163.com (C.W.); c420491898@163.com (Y.C.); spencerleo@163.com (Z.L.); 2 School of Life Science and Biopharmaceutics, Shenyang Pharmaceutical University, Shenyang 110016, China

**Keywords:** anti-tuberculosis, absolute configuration, DFT, ECD, X-ray diffraction, docking

## Abstract

A series of bedaquiline analogs containing H-bond donors were designed as anti-*Mycobacterium tuberculosis* drugs. A pair of diastereoisomers (*R/S*- and *S/S*-isomers) was selected from these designed compounds for synthetic and stereochemical research. The title compounds were synthesized from chiral precursors for the first time and the absolute configurations (ACs) were determined by electronic circular dichroism (ECD) with quantum chemical calculations. Moreover, a single crystal of the *S/S* compound was obtained for X-ray diffraction analysis, and the crystal structure showed high consistency with the geometry, confirming the reliability of ACs obtained by ECD analyses and theoretical simulation. Furthermore, the effect of stereochemistry on the anti-tuberculosis activity was investigated. The MICs of the *R/S*- and *S/S*-isomers against *Mycobacterium phlei 1180* are 9.6 and 32.1 μg·mL^−1^, respectively. Finally, molecular docking was carried out to evaluate the inhibitory nature and binding mode differences between diastereoisomers.

## 1. Introduction

The worldwide incidence of tuberculosis (TB) has increased substantially over the past decade due to *Mycobacterium tuberculosis* (MTB) [1]. Sirturo^®^ (bedaquiline), a diarylquinoline (DARQ) compound, is a new potent anti-tuberculosis drug which can block the proton pump of adenosine triphosphate synthase (ATPase) and subsequently disorder the energy metabolism of bacteria [2,3]. ATP synthase is a common enzyme in the membranes of eukaryotes and prokaryotes, mainly responsible for synthesis of ATP [4,5]. ATPase consists of lipophilic intramembrane region (F_0_, contains a- and c-subunit) and a more polar ATP-binding site (F_1_) [6,7]. Marc et al. proposed that the anti-tuberculosis activity of DARQ is based on interference with proton transfer between the a- and c-subunits. DARQ mimicks the conserved Arg-186 residue of the a-subunit and interacts in its place with the conserved Glu-61 of the c-subunit [8]. According to the literature, there are two stereogenic centers in DARQ-compounds which lead to four stereochemically different structures, namely the corresponding *R/S*, *S/R*, *S/S* and *R/R* stereoisomers. The *R/S* stereochemical feature of bedaquiline makes it more active and selective than other isomers; thence, it has been approved by FDA in 2012 as a part of combination therapy for multi-drug resistant TB [3,8,9,10].

**Scheme 1:**
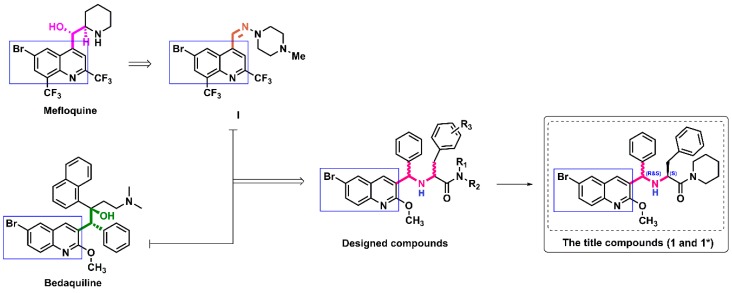
Design of bedaquiline analogs.

During the development of DARQ compounds, scientists paid much attention to the structure-activity relationship (SAR) of these compounds. An Indian group proposed to divide bedaquiline into four quarters [11]; subsequently, a lot of research was performed on modulating each sector to probe the biologically active motifs [12,13,14,15,16,17,18,19]. Recently, several researches have reported that some mefloquine-based compounds possess relatively potent activity against non-replicating persistent TB (MIC = 13 μM); and a remarkable similarity between mefloquine and bedaquiline was found when it was superimposed with bedaquiline replacing a hydroxyl group with a C-N double bond [20]. In mefloquine-based compounds, the derivative **1** demonstrated better or equal anti-TB activity to that of mefloquine. Our group has focused on the development of potent anti-tuberculosis drugs [21,22,23,24], and in our preciously studies, we have designed and synthesized bedaquiline analogies which possessed similar spatial molecular structures as bedaquiline. However, in the case of anti-tubercular activity studies, although most compounds demonstrated biological activity, the unsatisfactory result was that all showed lower inhibitory activities than the standard drugs. According to the structure activity relationship we have proposed, we assume that a lack of hydrogen bonding donors in the ligand could account for the weak activity of such compounds. In the present study, considering the importance of the chiral features in the bedaquiline molecule and H-bond donors in the ligand, “molecular hybridization” was adopted to design new quinoline derivatives which contain H-bond donors (the hydrogen atom of an amino group), and these were synthesized for the first time to develop anti-tuberculosis drugs (Scheme 1).

**Scheme 2:**
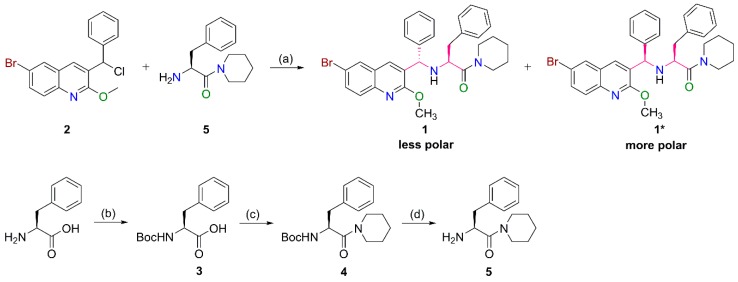
Synthesis of the title compound. *Reagents and Conditions*: (**a**) tetrabutylammonium iodide, K_2_CO_3_, acetonitrile, 82 °C, 6 h, column separation, 27.0%; (**b**) (Boc)_2_O, Na_2_CO_3_, water/tetrahydrofuran (10:1), rt., 12 h, 86.3%; (**c**) 4-methylmorpholine, isobutyl chloroformate, −15 °C, 30 min; piperidine, rt., 2 h, 91.0%; (**d**) HCl, rt., 2 h, 91.0%.

It is challenging to obtain optical pure compounds in a usual way. In our research, the stereogenic centers in the designed compound were constructed by introducing a chiral template to the racemic starting material (Scheme 2); therefore a pair of diastereoisomers would be generated, which could be separated into optically pure products by common column chromatography. Subsequently, absolute configuration determination of the obtained compound is required before further investigation. X-ray diffraction (XRD) can provide unambiguous elucidation of molecular structure and configurational assignments but requires a single crystal of the molecule [25]. As not every compound can provide high quality crystals and this limits XRD’s application range, electronic circular dichroism (ECD) is an ideal alternative for determining the absolute configuration of a molecule which has chromophores near the chiral center [26]. However, multiple chiral centers in the studied molecule would lead to a very complex curve, resulting in incorrect assignments. Fortunately, the broadest application of chiroptical methods in structural analysis has been remarkably facilitated by the development of methodology for *ab initio* predictions of chiroptical properties. With the aid of quantum-chemical calculations, one can determine the absolute configuration of compounds by judging whether the predicted findings for the chosen configuration match well with the measured curves [27,28].

The main objective of this work is to establish the relationship between presence of the hydrogen bonding donor in the designed compounds and anti-tuberculosis biological activity. Meanwhile, the stereostructural elucidation and effect of stereochemistry on anti-tuberculosis activities would be investigated during this study. In this paper, a pair of diastereoisomers of the designed compounds was selected as candidates for structural and anti-tuberculosis research. Firstly, the ACs of the two compounds were determined by experimental and theoretical ECD spectra. Furthermore, X-ray analysis of one isomer was preformed to confirm the stereostructure and gain deeper insight into the structural characteristics of the designed analogs. Finally, in vitro anti-tuberculosis activities of target compounds were evaluated, and molecular docking was carried out to explain the difference in inhibitory nature of designed diastereoisomers against a homology modeled ATP synthase enzyme.

## 2. Results and Discussion

### 2.1. Preparation of the Target Compounds

The target compounds were synthesized starting from 6-bromo-3-(chloro(phenyl)methyl)-2-methoxyquinoline (**2**) (Scheme 2) [13]. At first, synthesized chiral template (*S*)-2-amino-3-phenyl-1-(piperidin-1-yl)propan-1-one (**5**) was introduced to **2**, thus a mixture of diastereoisomers **1** and 1* which showed two distinguishable spots on TLC was generated. Common column chromatography enabled these to be separated into the less polar–**1** and more polar–1*.

### 2.2. Conformational Analyses of ***1*** and 1*

The conformational features of a molecule critically influence its physical and chemical properties. In the cases of **1** and 1*, the single bonds that connect three independent chromophores, may rotate at room temperature which can lead to conformational changes. As a result, the changes of the relative position of chromophores may markedly influence the shape of the ECD curves. Therefore, firstly, an accurate conformational analysis must be carried out to arrive at stable conformers of the title compounds.

**Figure 1:**
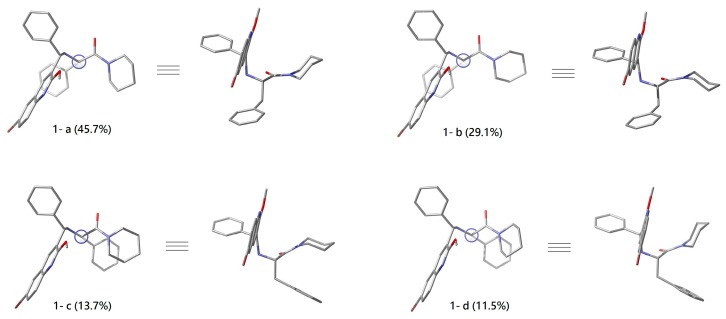
Relative stable conformers of **1**. All conformers are shown as “Newman projections” viewed along the C10-C13 bond axis.

A conformational search of **1** and 1* were performed in Spartan 14 using Monte Carlo searching together with MMFF [29,30] molecular mechanics calculations. Then full geometry optimizations of these obtained structures were employed by DFT/B3LYP/6-311++G(2d,p) [31,32,33,34] method. Finally, four stable conformers have been obtained for compounds **1** and 1*, respectively, which are shown in Figure 1 and Figure S1. Gibbs free energies, relative Gibbs free energies and Boltzmann weighting factors of conformers are presented in Table 1 and Table S1. In order to analyze the structural differences between conformations, Newman Projection was utilized to visualize the conformation of noncyclical molecules. Newman projections of the conformers were generated by viewing the molecules along the C10-C13 bond axis.

In the case of **1**, conformers **1**-**a** (45.7%), **1**-**b** (29.1%), **1**-**c** (13.7%) and **1**-**d** (11.5%) are significantly populated at room temperature. Newman projections of the conformers show that in conformers **1**-**a** and **1**-**b** the front quinoline ring is perpendicular to the back phenyl ring, causing C–H···π interactions; while in **1**-**c** and **1**-**d** the piperidine ring and back phenyl ring generally eclipse each other, which forces more repulsive interactions between the front and back attachments, creating more strain. It is noted that the slight difference between **1**-**a** and **1**-**b**, **1**-**c** and **1**-**d** was mostly focused on the “chair” conformer of piperidine ring, which may results in different intermolecular interactions. In terms of 1*, in 1*-**a** and 1*-**b** conformers, the piperidine ring and back phenyl ring close out each other. For 1*-**c** and 1*-**d**, two phenyl rings are located in one side, which lead to crowding. The differences between 1*-**a** and 1*-**b**, 1*-**c** and 1*-**d** are similar to those in the **1** conformers.

### 2.3. Determination of the Absolute Configurations of ***1*** and 1* by ECD

**Figure 2:**
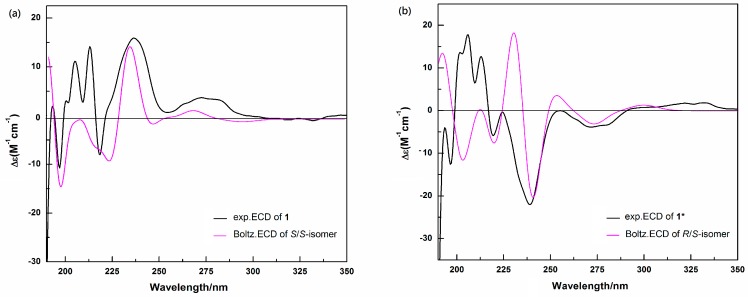
Experimental and calculated (TD-DFT/CAM-B3LYP/tzvp) ECD spectra of (**a**) **1** and (**b**) 1*.

The two stereogenic centers in the target compounds may lead to two possible absolute configurations: the *S/S* configuration (**1**) and *R/S* configuration (1*), due to the fact that the configuration of the introduced chiral source is already known as *S*-configuration and the other chiral carbon needs to be identified. To get a reliable stereochemical assignment of **1** and 1*, electronic circular dichroism (ECD) spectra provided by measured and quantum chemical calculation (TD-DFT/CAM-B3LYP/TZVP) were performed. The simulated ECD spectra for **1** and 1* which have been re-plotted with population weighting along with experimental spectrum are shown in Figure 2.

The Boltzmann-weighted ECD curves for the *S/S* configuration reproduced the sign and intensity of the measured negative Cotton Effect (CE) at 220 and 254 nm and positive CE at 206 nm, in excellent agreement with the experimental pattern of **1**. On the contrary, the simulated ECD spectrum for *R/S* was almost opposite to the experimental curve of **1**, while provided excellent agreement to the measured ECD bands of 1*. Notwithstanding some small discrepancies, this result supports the assignment of *S/S* configuration to **1** and *R/S* configuration to 1*, respectively.

**Figure 3:**
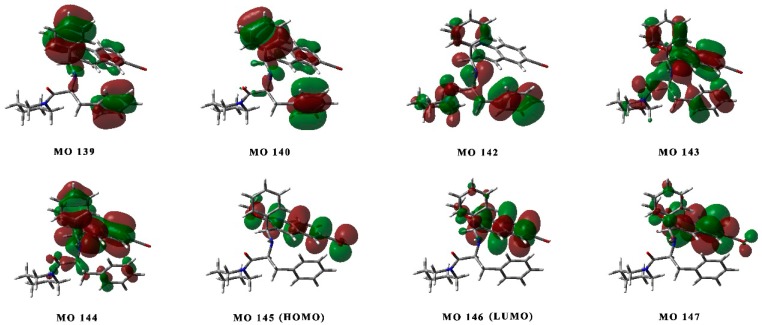
Molecular orbitals involved in the electronic transitions of **1**.

The origin of the CEs in ECD spectra of **1** and 1* could be explained by molecular orbital (MO) analysis at the same level as the ECD calculation. Figure 3 shows the MOs of **1** mainly involved in the electronic transitions used to assign the measured bands. As inferred from the MO analysis, the negative CE at 206 nm has contributions from the electronic transition from MO139, MO140 and MO142 to LUMO146 (LUMO = lowest unoccupied MO). The significant positive CE at 220 nm in the experimental spectrum mainly dominated by the transition from MO145 (HOMO = highest occupied MO) to MO147 involving the π-π* transition of quinoline ring. Moreover, the electronic absorption peak at 254 nm can be described by the transition from MO144 to LUMO146, which consists of π-π* from phenyl ring to quinoline ring. In the case of 1* (Figure S3), the CEs at 215 and 242 nm in ECD spectrum are dominated by the same transitions as the CEs at 206, 220 and 254 nm for 1*.

### 2.4. X-ray Crystal Structure Analysis of ***1***

**Figure 4:**
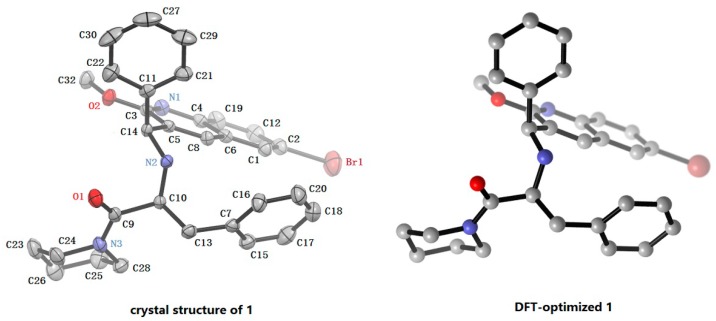
DFT-optimized and crystal structure of **1**, displacement ellipsoids are drawn at the 30% probability level.

Crystal of compound **1** was grown by slow evaporation of a mixed solution of ethyl acetate and petroleum ether (EtOAc:PE = 1:5) under ambient conditions, and a crystal suitable for crystallographic analysis was obtained. The measured values reveal that **1** possesses monoclinic crystal system having P21 21 21 space group [unit cell: a = 9.2390(3) Å, b = 15.4237(5) Å, c = 19.6921(6) Å, α = 90.00°, β = 90.00°, γ = 90.00° and V = 2806.12(15) Å^3^]. ORTEP diagram of the molecular structure and the atomic numbering schemes of title compound are shown in Figure 4. The hydrogen atoms are omitted for clarity. The crystallographic and refinement data are shown in Table 2.

**Figure 5:**
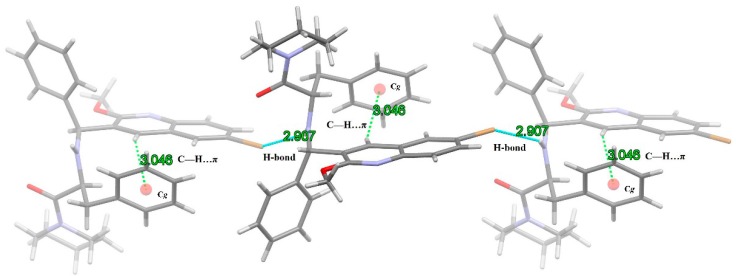
Part of the packing diagram of **1** showing the formation of intramolecular hydrogen bonding and C-H…π interaction. C*g* stands for the centroid of the ring.

To obtain deeper insight into the structure characteristics of the designed compound, an X-ray structure analysis was performed. As depicted by the crystal structure in Figure 4 the absolute configuration at the stereogenic center in the target compound could be determined unambiguously: the α-carbon relative to the quinoline moiety is *S* and the other chiral carbon is *S*, is agreement with the ECD result. The piperidine ring exists in a ‘chair’ conformation and the torsion angle of C(24)-C(28)-C(9)-C(3) is 3.43°. Moreover, the X-ray crystal data reveals that each molecule interacts with two other molecules, and forms a trimer via N(2)-H(2)···Br(1)i (i = −1/2 − x, 2 − y, 1/2 + z) and Br(1)···H(2)-N(2)ii (ii = −1/2 − x, 2 − y, 1/2 + z) hydrogen bonds (Table 3). In addition to the afore-described interactions, the crystal can be stabilized by a weak C-H···π interaction between the substituted quinoline and the plane of the π-electron system of benzene too (Figure 5).

Additionally, the electronic charge on an atom obtained by Mulliken population analysis determines the bonding capability [35]. The Mulliken atomic charges on the component atoms of the target molecule have been calculated by B3LYP/6-311++G(2d,p). The theoretical Mulliken atomic charges of Br(1) and H(2) are −0.180 and 0.240 e, respectively. Therefore, Mulliken atomic charges explain the presence of the Br(1)···H(2) H-bond.

The crystal structure of compound **1** was compared with the DFT-optimized structure. Among all the conformers of **1**, conformer **1**-**a** was in accord with the crystalline structure. This was consistent with our designed structure of compound **1**, which illustrated that the molecular structures and absolute configuration of designed compounds were correct. Some selected experimental and calculated geometry parameters for **1** are listed in Table 4.

The experimental bond lengths of pivotal bonds, such as Br1–C2, N2–C10, N2–C14, N3–C9, O1–C9, O2–C3, C5–C14, C9–C10, C10–C13 and C11–C14 appear at 1.861(4) Å, 1.449(5) Å, 1.472(5) Å, 1.347(6) Å, 1.204(5) Å, 1.332(5) Å, 1.518(5) Å, 1.546(6) Å, 1.543(6) Å and 1.498(6) Å, respectively, which agrees nicely with the theoretical values (with differences up to 0.060 Å). The measured bond angles for C3–O2–C32, Br1–C2–C12, C10–N2–C14, N3–C9–C10, N2–C10–C13 and C5–C14–C11 are 117.1(4)°, 119.6(3)°, 114.4(3)°, 117.6(4)°, 109.8(3)° and 112.2(3)°, respectively (differences of up to 2.0° only). As expected, most of the calculated geometry parameters for the title compound are close to the X-ray data.

### 2.5 Anti-Tuberculosis Activity

In the anti-tubercular activity studies, both compounds **1** and 1* possessed biological activity, but with MICs of 9.6 and 32.1 μg·mL^−1^, respectively, it was observed that the inhibitory activity of 1* was better than that of **1**. This is consistent with our hypothesis that the inhibitory activity of the *R/S* isomer is better than that of the *S/S* stereoisomer.

### 2.6 Docking Analysis

**Figure 6:**
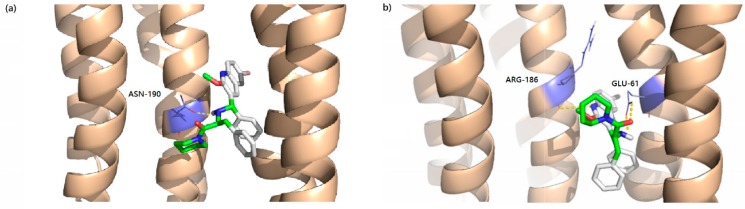
The ATPase active site in complex with compound **1** (**a**) and 1* (**b**).

To evaluate the nature if the inhibitory activity against tuberculosis and the binding differences of the designed compounds, a molecular docking study was performed. As previously mentioned, the target of diarylquinoline (DARQ) compounds is ATPase which responsible for the synthesis of ATP. Site-directed mutations and biological information indicate that the binding site of DARQ occurs on the a- and c-subunits of the F_0_ portion of ATPase [8], which is encoded by the atpB (sequence P63654) and atpE (sequence Q10598) genes [8,36]. ATPase is a common enzyme in the membranes of eukaryotes and prokaryotes, and like membrane proteins, it is very difficult to crystallize [4,5]. An accurate structure of ATP synthase is essential for precise docking results, but to date, there is no available structure for mycobacterial ATPase, its subunits, or the binding site of DARQ in this enzyme; fortunately, homology modeling combined molecular dynamics could offer a satisfactory structure from known structurally homologous proteins. The template protein is the F_0_-region of *Escherichia coli* ATP synthase which PDB code is 1C17. The homology model based on the alignment (Figure S4) of *Mycobacterium tuberculosis* and *Escherichia coli* sequences was carried out by Modeller 9.14 package [37]. The modeling structure and literature suggest the interaction site of DARQ is located on the gap between the a-subunit and c-subunit. In order to exclude bad implausible van der Waals contacts and to obtain the most advantageous conformation for the constructed models of ATPase of *Mycobacterium tuberculosis*, a molecular dynamics (MD) simulation was conducted using the Thinker [38] minimizer. The best interacting a- and c-subunit was chosen as a reasonable geometry for the following docking simulation (Figure S5). According to the modeling structure of ATPase and the literature, the bonding pocket was identified nearby the Arg-186 of a-subunit and Glu-61 of c-subunit which controls the transition of protons from the a-subunit to the c-subunit, and within the grid size of 35Å × 35Å × 35Å. The ligand was prepared for docking by pre-optimizing at DFT/B3LYP/6-311++G(2d,p) level. Molecular docking calculations of the title compound with ATPase were performed on the AutoDock-Vina software [39] using the Lamarckian Genetic Algorithm (LGA) available in AutoDock, and the proposed binding modes for **1** and 1* are illustrated in Figure 6.

In the docking complex of **1** and ATPase, the amine hydrogen forms a key H-bond with the residue Asn-190 of the a-subunit in the binding pocket. For 1* complex, the amine hydrogen atom and the oxygen atom of amide group form key H-bonds with the Glu 61 residue of the c-subunit, moreover, another hydrogen bond exists between the nitrogen atom of the quinoline ring and the Arg-186 moiety of the a-subunit. As previously mentioned, the interaction between inhibitor and Arg-186 (a-subunit), Glu-61 (c-subunit) can inhibit the activity of ATPase. Obviously, the *R/S*-isomer would show better anti-tuberculosis activity than *S/S*-isomer, which is due to the stereogenic center, so different biological activities were detected. Furthermore, the interaction between ATPase and designed compounds is of particular interest, as the latter plays the part of hydrogen bonding donor which confirms our hypothesis that H-bond donation is an important feature of diarylquinolines derivatives.

## 3. Materials and Methods

### 3.1. General Information

IR spectra with 1.0 cm^−1^ resolution were recorded on an IFS-55V IR spectrometer (Bruker, Ettlingen, Germany) in the 4000–400 cm^−1^ region using the KBr pellet technique. ^1^H-NMR spectra (600 MHz) and ^13^C-NMR spectra (150 MHz) were recorded at 293.4 K on a Bruker AVANCE-600 NMR spectrometer, with tetramethylsilane (TMS) as an internal standard and CDCl_3_ as solvent. Mass spectroscopy was performed on a Hewlett-Packard 1100 LC/MSD spectrometer (Hewlett-Packard, Palo Alto, CA, USA). ECD spectra were recorded on a CD-2095 spectropolarimeter (JASCO, Tokyo, Japan). X-ray data of a selected crystal with dimensions of 0.32 × 0.22 × 0.15 mm, picked up under microscope; and then fixed on a glass tip supported by a copper pin and a magnetic base, were recorded by a Bruker P4 X-diffractometer, equipped with graphite monochromatic Mo-Kα radiation (λ = 0.71073 Å) for data collection at 293 K. The data was collected by APEX2 [40]; the structure solution was performed using SHELXS-97 [41]; SHELXL-97 [42] for cell refinement and Mercury 2.3 [43] for the visualization of structure. CCDC-1419767 (for **1**) contains the supplementary crystallographic data for this paper. These data can be obtained free of charge via http://www.ccdc.cam.ac.uk/conts/retrieving.html (or from the CCDC, 12 Union Road, Cambridge CB2 1EZ, UK; Fax: +44 1223 336033; E-mail: deposit@ccdc.cam.ac.uk).

### 3.2. Materials

Tetrahydrofuran and acetonitrile (Aldrich, Co. Ltd., Shanghai, China) were purchased and used after drying with molecular sieves 4Å. l-Phenylalanine, di-*tert*-butyl dicarbonate, 4-methylmorpholine, piperidine, tetrabutylammonium iodide, sodium iodide, potassium carbonate and isobutylchloroformate were also commercial products (Wako Co. Ltd., Tokyo, Japan). All the reagents were of analytical grade. For TLC analysis, precoated plates of silica gel 60 F254 were used. Spots were visualized with UV light. 3.3. Synthesis of **1** and 1*.

#### 3.2.1. General Procedure for the Synthesis of **5**

L-Phenylalanine (1.65 g, 10.0 mmol) and di-*tert*-butyl dicarbonate (10.91 g, 50.0 mmol) were added to a solution of water/tetrahydrofuran (100 mL, 10:1) at 0 °C and sodium carbonate (0.53 g, 5.0 mmol) was added to the mixture. After stirring for 12 hours at room temp., 10% hydrochloric acid (100 mL) was dropped into the reaction mixture, the pH adjusted to 2 and then extracted with ethyl acetate (100 mL × 3). The organic phase was then washed by saturated brine, dehydrated with anhydrous sodium sulfate and concentrated at reduced pressure, and the residue was recrystallized by 50% ethyl acetate in petroleum ether to afford **3** (2.29 g, 86.3% yield). To a solution of **3** (2.29 g, 8.6 mmol) and 4-methylmorpholine (0.87 g, 8.6 mmol) in dried tetrahydrofuran (50 mL) at −15 °C was added isobutylchloroformate (1.68 g, 10.3 mmol) diluted with dry tetrahydrofuran (10 mL). After stirring for 30 min at −10 °C, piperidine (0.88 g, 10.3 mmol) was added into the mixture that was then stirred for 2 h at room temp. Then, the mixture was treated by water and exacted by ethyl acetate (100 mL × 3), the organic layer was successively washed with 10% sodium carbonate solution (30 mL × 2), 0.1 mol/L hydrochloric acid (30 mL × 2) and saturated brine (30 mL × 2). After that, the combined organic phase was dried over anhydrous sodium sulfate and concentrated under reduced pressure, and the residue was recrystallized from 50% ethyl acetate in petroleum ether to afford **4** (2.60 g, 91.0% yield). Compound **4** (2.60 g, 7.8 mmol) was dissolved in methanolic hydrochloride solution (50 mL), stirred for 2 h at room temp and then concentrated under reduced pressure to afford **5** (1.65 g, 91.0% yield).

#### 3.2.2. General Procedure for the Synthesis of **1** and 1*

Compound **2** (0.26 g, 0.7 mmol) and 5 (1.65 g, 7.1 mmol) was dissolved in acetonitrile (50 mL), and potassium carbonate (0.12 g, 0.8 mmol) and tetrabutylammonium iodide (0.01 g, 0.04 mmol) were added to the above solution. After stirring for 6 hours at 82 °C, the reaction mixture was quenched with water and then extracted with ethyl acetate. The organic phase was washed by saturated brine (30 mL × 2), and then the combined organic phase was dried over anhydrous sodium sulfate. After concentrating under reduced pressure, the residue was separated on silica gel using a petroleum ether- ethyl acetate gradient (from 100:1 → 10:1 *v*/*v*) for purification, to give a white powder consisting of 0.15 g (39.0%) of **1** and 0.07 g (19.0%) of 1*.

*(S)-2-(((S)-(7-Bromo-3-methoxynaphthalen-2-yl)(phenyl)methyl)amino)-3-phenyl-1-(piperidin-1-yl)propan-1-one* (**1**). White solid ${\left[\alpha \right]}_{D}^{25}$ = +45.5 (*c* = 1, CH_2_Cl_2_). IR (KBr pellets)/cm^−1^: 3306.3 (N–H); 3060.0, 3028.0 (Ar-H), 2918.7, 2854.0 (OCH_3_); 1645.9 (C=O); 1599.2 (ArC=C); 1466.5, 1436.7 (OCH_3_); 953.2 (Ar–H); 555.0 (C-Br). ^1^H-NMR (600 MHz, CDCl_3_): *δ* 1.46–1.74 (6H, m), 2.76–2.96 (4H, m), 3.08 (1H, s), 3.52–3.54 (1H, t, *J* = 4.8 Hz), 3.65–3.71 (2H, dd, *J* = 6, 36 Hz), 3.95 (3H, s), 5.04 (1H, s), 7.20–7.22 (1H, m), 7.23–7.25 (2H, t, *J* = 7.2 Hz), 7.27–7.28 (2H, d, *J* = 7.2 Hz), 7.36–7.37 (2H, d, *J* = 7.2 Hz), 7.38–7.40 (2H, t, *J* = 7.2 Hz), 7.40–7.42 (1H, m), 7.49–7.50, 7.70–7.71 (1H, dd, *J* = 6, 1.8 Hz), 7.58–7.63 (2H, m), 7.76–7.78 (1H, d, *J* = 12 Hz). ^13^C-NMR (150 MHz, CDCl_3_): *δ* 172.51, 160.77, 144.16, 143.90, 142.39, 138.62, 134.69, 132.02, 130.00, 129.77, 129.47, 129.05, 128.46, 128.34, 128.11, 127.80, 127.33, 126.72, 126.68, 126.14, 116.87, 58.29, 57.67, 53.50, 46.14, 43.42, 40.51, 29.72, 26.18, 25.82, 24.48. MS (ESI(+)): *calcd.* for C_31_H_32_BrN_3_O_2_ [M + H]^+^ *m*/*z* 558.17, found 558.2; *calcd.* for C_31_H_32_BrN_3_O_2_ [M + 2+ H]^+^ *m*/*z* 560.17, found 560.2.

*(S)-2-(((R)-(7-Bromo-3-methoxynaphthalen-2-yl)(phenyl)methyl)amino)-3-phenyl-1-(piperidin-1-yl)propan-1-one* (1*). White solid. ${\left[\alpha \right]}_{D}^{25}$ = −45.1 (*c* = 1, CH_2_Cl_2_). IR (KBr pellets)/cm^−1^: 3306.3 (N-H); 3059.9, 3026.0 (Ar–H), 2921.1, 2851.6 (OCH_3_); 1632.8 (C=O); 1569.1 (ArC=C); 1491.4, 1463.2 (OCH_3_); 924.0 (Ar–H); 570.2 (C–Br). ^1^H-NMR (600 MHz, CDCl_3_): *δ* 1.37–1.51 (6H, m), 2.58 (1H, s), 2.75–3.02 (4H, m), 3.46–3.55 (2H, dd, *J* = 6 Hz, 36 Hz), 3.63–3.67 (1H, t, *J* = 4.8 Hz), 4.01 (3H, s), 5.08 (1H, s), 7.16 (1H, m), 7.17–7.18 (1H, m), 7.20–7.23 (2H, t, *J* = 7.2 Hz), 7.24–7.25 (2H, d, *J* = 7.2 Hz), 7.27–7.28 (2H, d, *J* = 7.2 Hz), 7.30–7.33 (2H, t, *J* = 7.2 Hz), 7.41 (1H, dd, *J* = 2.0, 56.8 Hz), 7.68 (2H, d, *J* = 36.8 Hz,), 8.22 (1H, s). ^13^C-NMR (150 MHz, CDCl_3_): *δ* 171.33, 150.07, 143.13, 142.87, 140.15, 137.00, 134.18, 132.38, 131.01, 129.23, 128.51, 127.45, 127.28, 127.22, 127.19, 126.26, 125.70, 125.45, 125.28, 124.01, 115.95, 57.05, 55.00, 52.51, 44.81, 41.95, 40.10, 28.68, 24.69, 24.51, 23.25. MS (ESI(+)): *calcd.* for C_31_H_32_BrN_3_O_2_ [M + H]^+^ *m*/*z* 558.17, found 558.2; *calcd.* for C_31_H_32_BrN_3_O_2_ [M + 2+ H]^+^ *m*/*z* 560.17, found 560.2.

### 3.3. Computational Details

#### 3.3.1. Quantum Chemical Calculations

The exhaustive conformational searching was performed by Spartan 14 program with MMFF [29,30] molecular mechanics force field for the title compounds. In this step, the conformers within 5 kcal/mol energy window were re-optimized at Becke-3-Lee-Yang-Parr (B3LYP) supplemented with the standard 6-311++G(2d,p) [31,32,33,34] basis set by Gaussian 09 [44] software package. Gibbs free energies were also calculated at the same level and frequency calculations based on the previously optimized geometries in order to ensure the minimum energy of the structure. The Boltzmann weighting factor *P_i_*:

(1) $$P_{i}=\frac{\exp(-G_{i}/RT)}{\sum_{j}\exp(-G_{j}/RT)}\times 100\%$$

where *G_i_*, *R* and *T* stand for the Gribbs free energy, gas constant and absolute temperature (298 K). Then, redistributed energy values according to the Boltzmann distribution, regardless of the conformers which possess inappreciable contribution for conformational equilibrium, permitting define the stable conformers for further investigation. TD-DFT/CAM-B3LYP/TZVP under PCM model was employed to calculate excitation energy (denoted by wavelength in nm) and rotatory strength R. ECD curves were calculated based on rotatory strengths using half bandwidth of 0.16 eV with conformers by Specdis. The spectra were constructed based on the Boltzmann-weighting according to their population contribution.

#### 3.3.2. *Mycobacterium tuberculosis* ATPase Structural Prediction

The amino acid sequence of *Mycobacterium tuberculosis* ATPase (P9WPV7, P9WPS1) obtained from the Swiss-Prot TrEMBL database was used to search for the templates using the program BLAST [45] in the homology modeling method. *Escherichia coli* ATP synthase (PDB code: 1C17 [46]) displays the highest sequence identity to *Mycobacterium tuberculosis* ATPase in Protein Data Bank (PDB) [47]. The sequences of template and target protein have been aligned by ClustalW package [38] (Figure S1), and an initial estimate of the 3D structure was subsequently generated by using an *E. coli* model as a template by Modeller 9.14 [47]. In order to have a stable structure of *Mycobacterium* *tuberculosis* ATPase, the homology modeling protein was locally optimized with 1000 cycles of a parallel conjugate gradient minimizer based on an optimized version of Tinker minimizer [48]. Finally, the refined model was evaluated by several programs: Procheck [49], Verify_3D [50,51], and Errat [52], which belong to the structure analysis-validation (SAV) online server sponsored by the UCLA-DOE Institute for Genomics and Proteomics. All the evaluated values by SAV illustrated that the final refined model meets the requirements of protein for docking.

#### 3.3.3. Molecular Docking 

Molecular docking involves fitting a protein and ligand together in a favorable conformation to form a complex. Structural information from such a complex may help to clarify the inhibitory nature between inhibitor and enzyme. The AutoDock-Vina program [39] was used to identify the binding mode of compound **1** and 1* with *Mycobacterium tuberculosis* ATPase. The compounds were allowed to dock anywhere in a 35 Å × 35 Å × 35 Å volume centered around Glu-61 of c-subunit, with the Lamarckian genetic algorithm (LGA). The maximum numbers of energy evaluation and generation were set to 250,000 and 27,000, respectively. A total of 20 separate docking runs were performed with the initial population of 20 individuals, and the lowest energy structure from each run was retained. Final docked conformations were clustered by using a tolerance of 1.0 Å root mean square deviation (RMSD).

### 3.4. Antimycobacterial Test for ***1*** and 1*

The test stain (*Mycobacterium phlei 1180*) was obtained the China General Microbiological Culture Collection Center (Beijing, China). *M. phlei* stock cultures were maintained on Lownstein-Jenson slants whilst the working cultures were incubated in a defined medium with the initial pH 7.4–7.6 (100 mL) containing tryptone 1 g, beef extract 0.5 g, glycerol 0.5 g and NaCl 0.5 g. A series of broth tubes containing diluted chemical synthetic compound concentrations were prepared and inoculated with a 48 h liquid culture of *Mycobacterium phlei 1180*. The maximum concentration of compounds for biological test is 1 mg·mL^−1^, and then a serial dilution of the compounds were made. The final drug concentrations test were 0.01−1000.0 μg·mL^−1^. Then the tubes were incubated at 37 °C for 20 h. The MIC was defined as the lowest concentration that prevented the mycobacterial growth and their inhibition against other strains is test by naked eye. Rifampicin was chosen to be the standard drug and the MIC was 10.0 μg·mL^−1^. The MIC range of bedaquiline against *M. phlei* is 0.03−0.13 μg·mL^−1^ [53].

## 4. Conclusions

In conclusion, we have synthesized for the first time two chiral analogs of bedaquiline which contain H-bond donors. The ACs of the isomers were determined by experimental and theoretical ECD spectra, and X-ray structures analyses further validated the assignments of AC. Furthermore, the test for the anti-TB activity has demonstrated that *R/S*-isomer is better than the *S/S*-isomer. Subsequently, the target compound was docked into the pocket to explain the nature of the inhibitory activity and binding mode differences between diastereoisomers. The results revealed that the *R/S*-isomer could form key H-bond interactions with the Glu-61 residue of the c-subunit, and thus block proton transfer. Therefore, chiral features and hydrogen bonding of the ligand play an important role in the activity against *Mycobacterium tuberculosi*s.

## Figures and Tables

**Table 1:** Gibbs free energies (*G*), relative Gibbs free energies (Δ*G*) ^a^ and Boltzmann weighting factor (*P* %) ^b^ of compound **1** conformers by using the DFT/B3LYP/6-311++G(2d,p) method.

Conf.	*G* (kcal/mol)	△*G* (kcal/mol)	*P_i_*%
**1-a**	−2566110.908	0	45.7
**1-b**	−2566110.645	0.263	29.1
**1-c**	−2566110.203	0.705	13.7
**1-d**	−2566110.099	0.809	11.5

^a^ related to the most stable conformer, ^b^ Boltzmann weighting factor (*P_i_*%) based on Δ*G*.

**Table 2:** Crystal data and refinement details for compound **1**.

Empirical Formula	C_31_H_32_BrN_3_O_2_
Formula weight	558.5
CCDC number	1419767
Temperature (K)	296
Crystal size	0.32 × 0.22 × 0.15
Crystal color	colourless
Crystal system	monoclinic
Space group	P 21 21 21
a (Å)	9.2390(3)
b (Å)	15.4237(5)
c (Å)	19.6921(6)
α (°)	90.00
β (°)	90.00
γ (°)	90.00
V (Å^3^)	2806.12(15)
Z	4.00
Theta (max)	25.65
R1, wR^2^	0.0600, 0.1987
Data completeness	1.75/0.99
Max. and min. transmission	0.749, 0.741
Wavelength	0.71073

**Table 3:** Hydrogen bond geometry (Å, °) for crystal **1**.

D-H···A	d(D-H)	d(H…A)	d(D…A)	< (DHA)
N(2)-H(2)···Br(1)i	0.86	2.907	3.871	149.03
Br(1)···H(2)-N(2)ii	0.86	2.907	3.871	149.03

Symmetry codes: (i) −1/2 − x, 2 − y, 1/2 + z; (ii) −1/2 − x, 2 − y, 1/2 + z.

**Table 4:** Selected experimental and calculated geometry parameters for compound **1**.

Bond Distances (Å)	Exp.(1)	Cal.(1-a)	Diff.	Bond Angle (°)	Exp.(1)	Cal.(1-a)	Diff.
Br(1)–C(2)	1.861(4)	1.921	0.060	Br(1)–C(2)–C(12)	119.6(3)	118.6	−0.1
C(3)–O(2)	1.332(5)	1.351	0.019	N(2)–C(10)–C(13)	109.8(3)	110.3	0.5
C(5)–C(14)	1.518(5)	1.521	0.003	N(3)–(9)–C(10)	117.7(4)	119.7	2.0
C(9)–O(1)	1.204(5)	1.226	0.022	C(3)–O(2)–C(32)	117.1(4)	117.3	0.2
C(9)–N(3)	1.347(6)	1.365	0.018	C(5)–C(14)–C(11)	112.2(3)	112.2	0.0
C(9)–C(10)	1.546(6)	1.551	0.005	C(10)–N(2)–C(14)	114.1(3)	114.4	0.3
C(10)–N(2)	1.449(5)	1.457	0.008				
C(10)–C(13)	1.543(6)	1.551	0.008				
C(11)–C(14)	1.498(6)	1.526	0.028				
C(14)–N(2)	1.472(5)	1.476	0.004				

## Supplementary Materials

Supplementary materials can be accessed at: http://www.mdpi.com/1420-3049/21/7/875/s1.
