# Supplementary material for: Design and Stereochemical Research (DFT, ECD and Crystal Structure) of Novel Bedaquiline Analogs as Potent Antituberculosis Agents
Source: Molecules. 2016 Jul 4;21(7):875. doi: 10.3390/molecules21070875 (PMC6274456; doi:10.3390/molecules21070875)
Supplement: Supplementary file 1 [file molecules-21-00875-s001.pdf]

Supplementary Materials: Design and Stereochemical Research (DFT, ECD and Crystal Structure) of Novel Bedaquiline Analogs as Potent Antituberculosis Agents

Yiding Geng, Linwei Li, Chengjun Wu, Yumeng Chi, Zhen Li, Wei Xu and Tiemin Sun

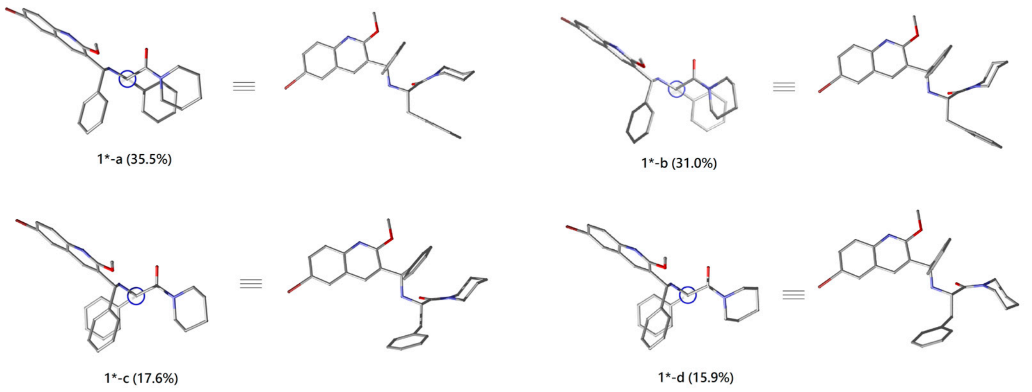

Figure S1. Relative stable conformers of **1***. All conformers are shown as "Newman Projections" by viewing along the C10-N13 bond axis.

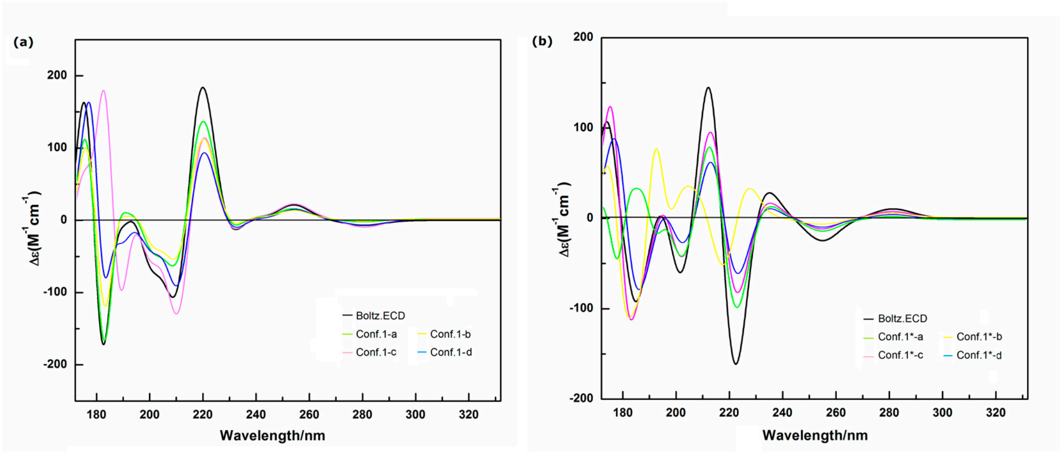

Figure S2. Calculated ECD spectra of individual conformers of (**a**) **1** and (**b**) **1***.

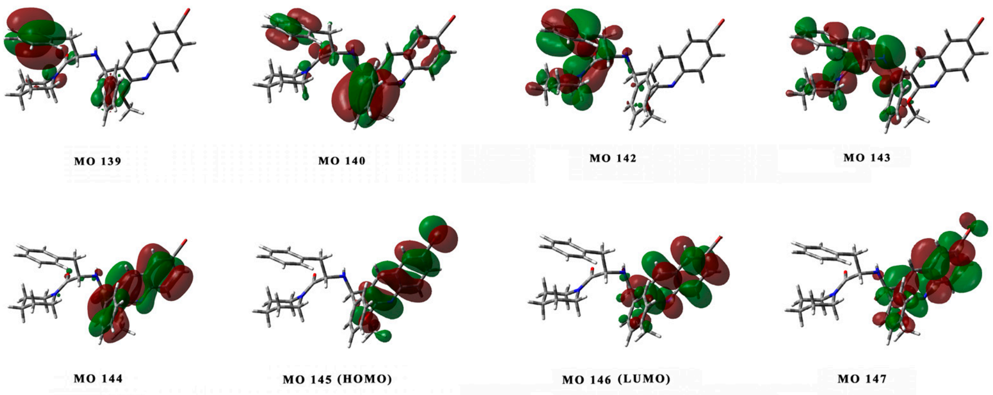

Figure S3. Molecular orbitals involved in the electronic transitions of **1***.

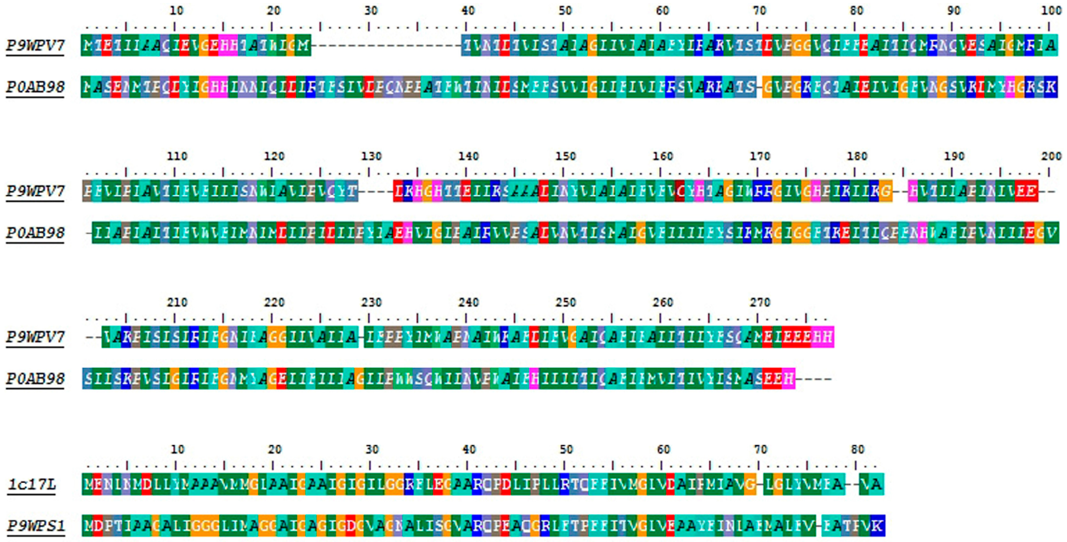

Figure S4. Alignments of the a-subunits (atpB gene) above and c-subunits (atpE gene) below of *Mycobacterium tuberculosis* and *Escherichia coli*. Sequence numbering in the a-subunits is different (upper numbers are for *Mycobacterium tuberculosis* lower numbers for *Escherichia coli*) The c-subunit sequence numbering is the same for these species.

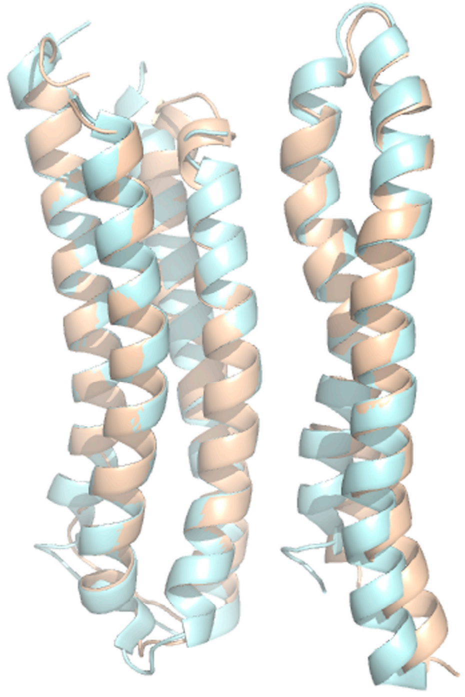

Figure S5. Superimpose for a- and c-subunits of *Escherichia coli* (carbon atoms indicated in blue) and homology model of *Mycobacterium tuberculosis* (carbon atoms indicated in brown).

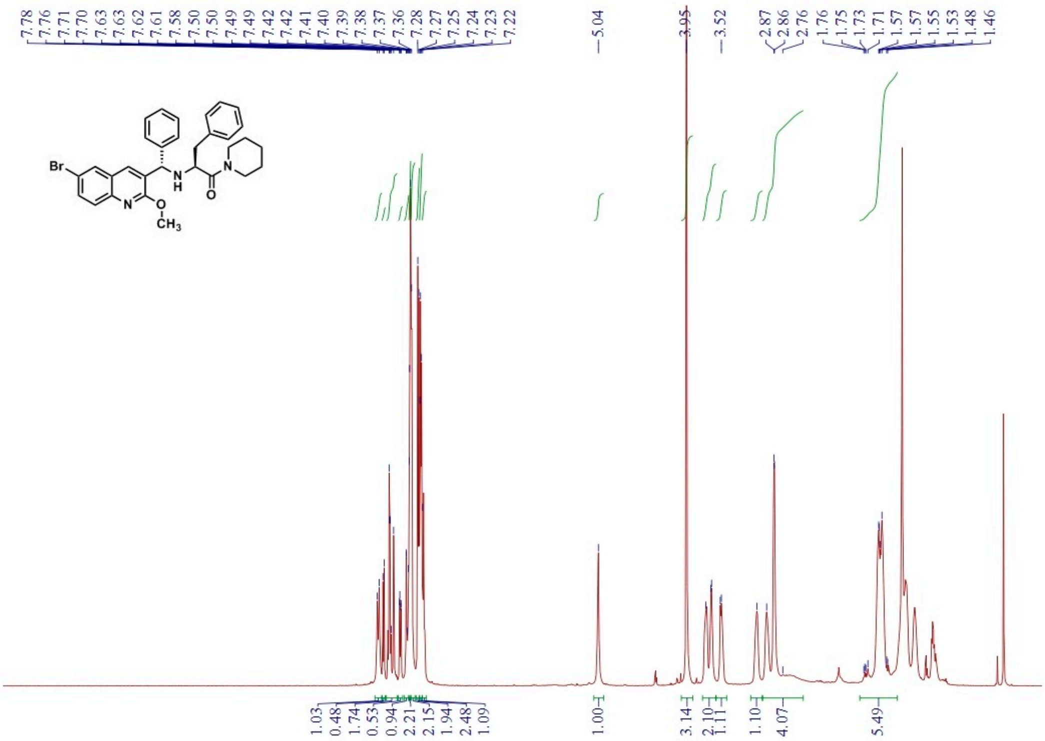

Figure S6. ^{1}H-NMR spectrum of compound **1**.

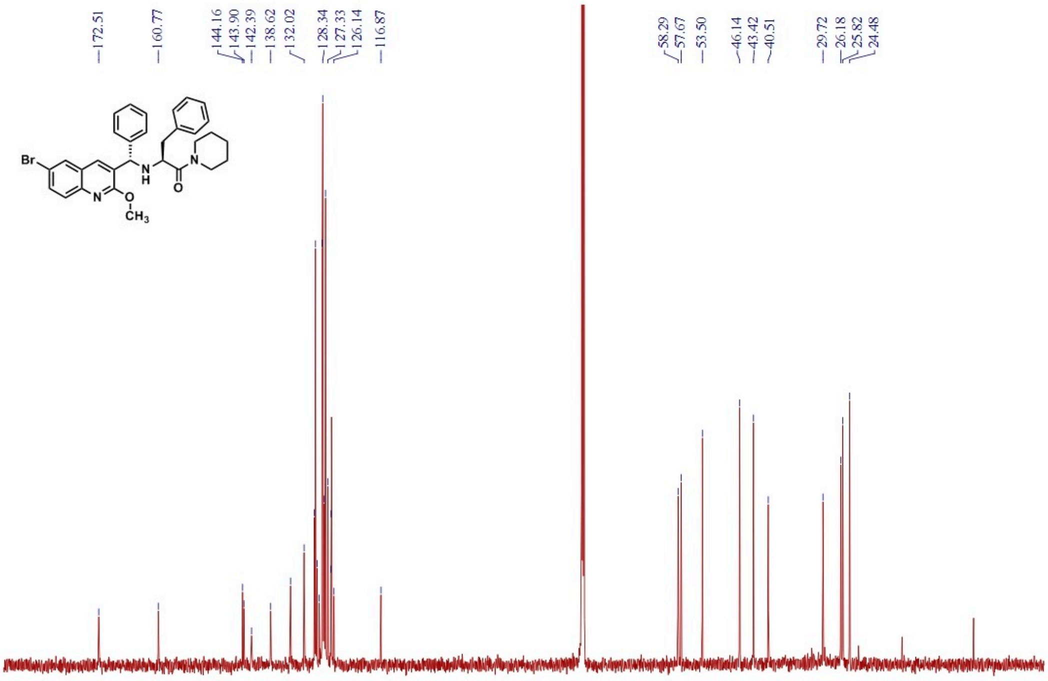

Figure S7. ^{13}C-NMR spectrum of compound **1**.

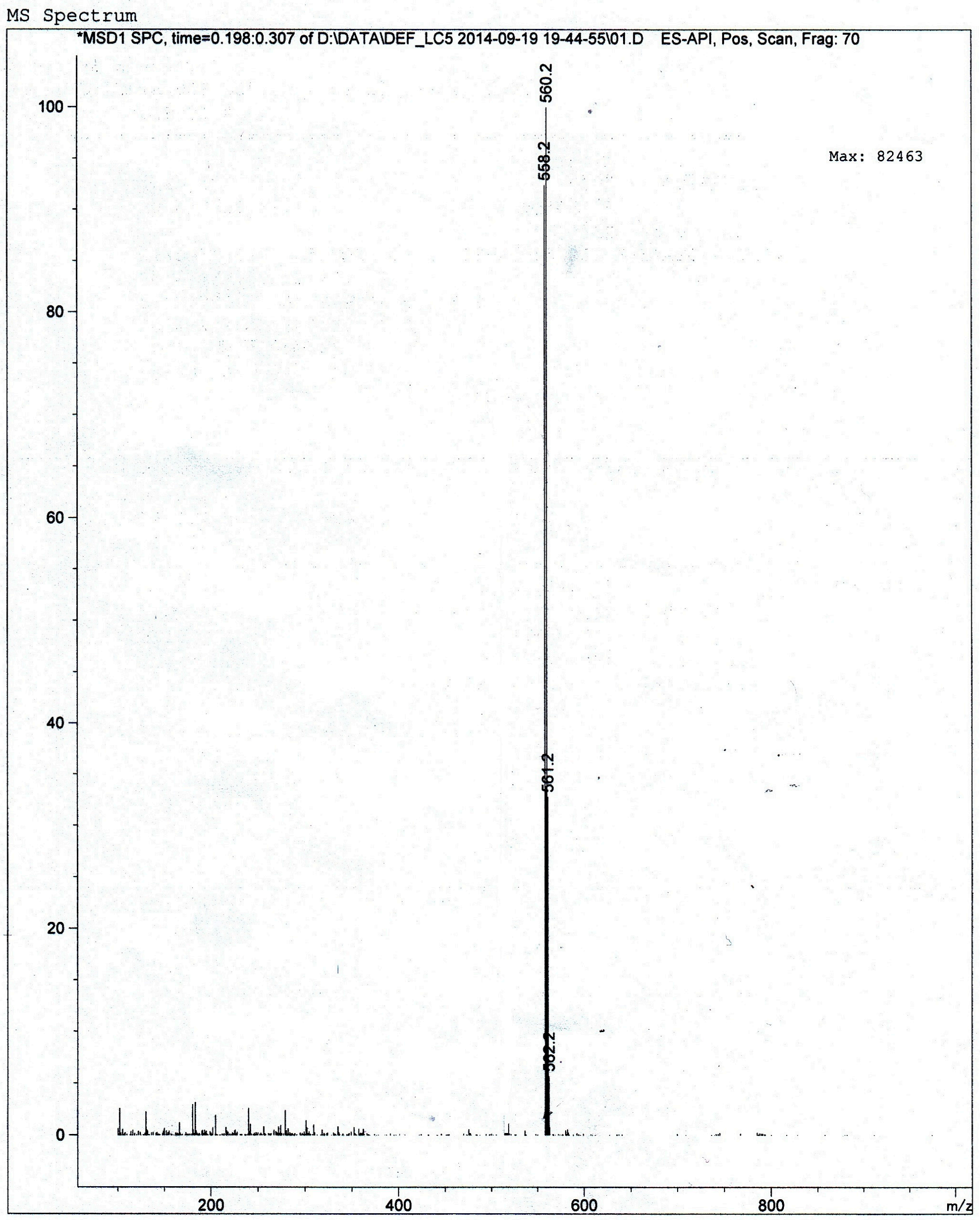

Figure S8. MS spectrum of compound **1**.

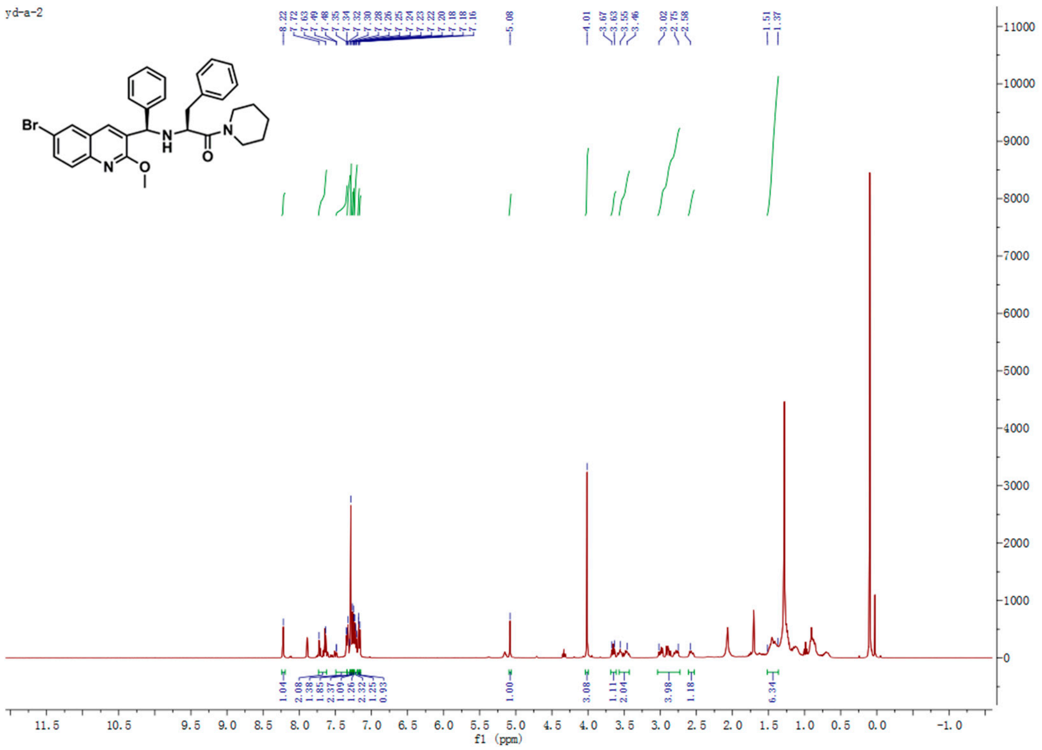

Figure S9. ^{1}H-NMR spectrum of **1***.

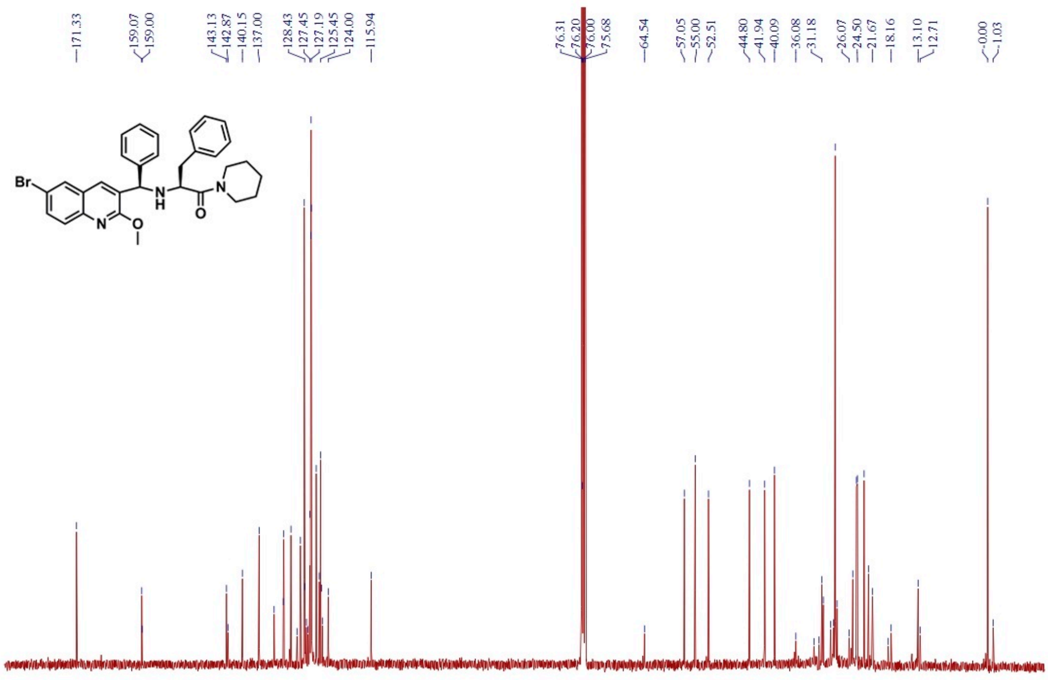

Figure S10. ^{13}C-NMR spectrum of **1***.

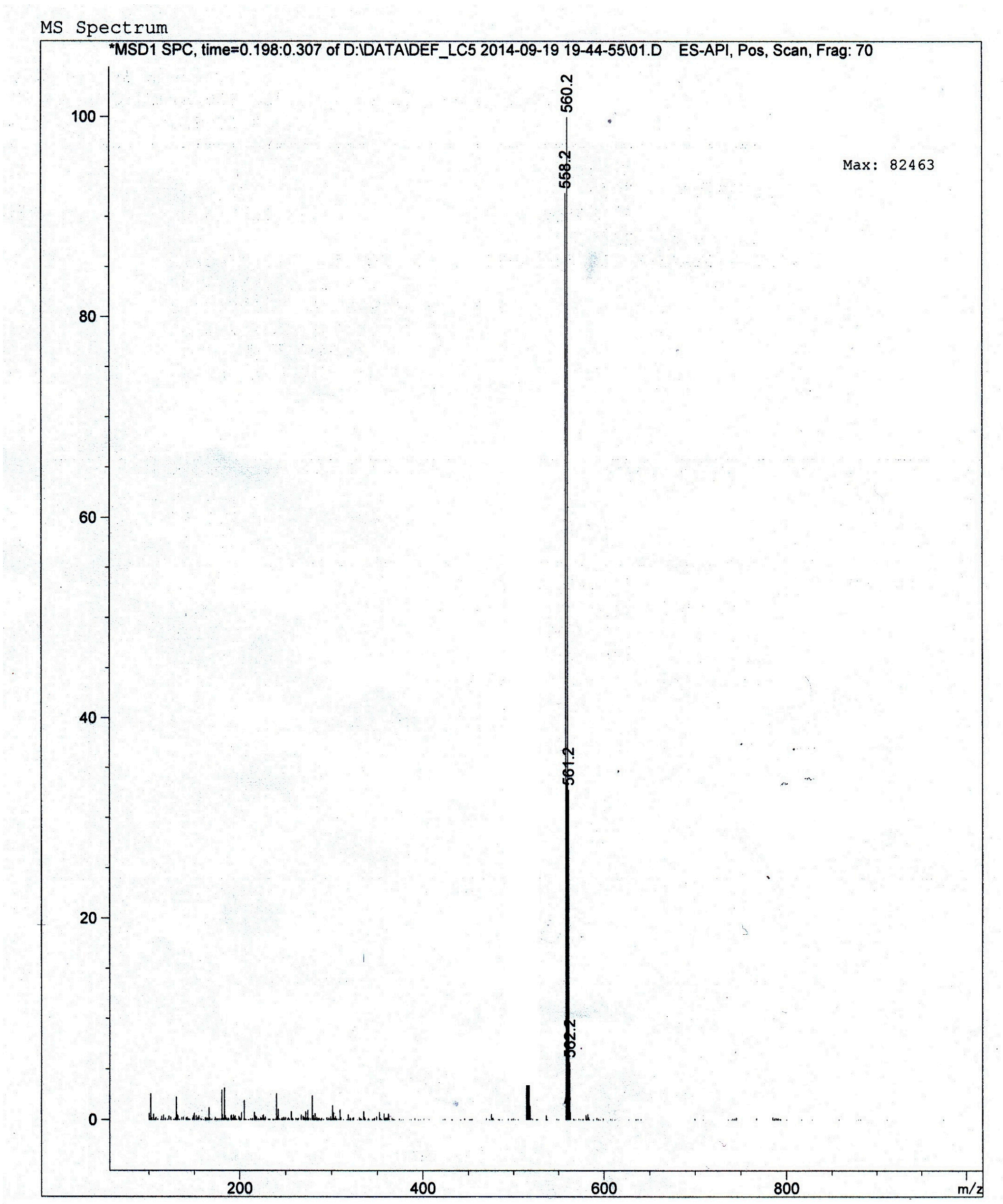

Figure S11. MS spectrum of **1***.

Table S1. Gibbs free energies (*G*), relative Gibbs free energies (Δ *G*) [a] and Boltzmann weighting factor (*P*%) [b] of compound **1*** conformers by using the DFT/B3LYP/6-311++G(2d,p) method.

Conf.	*G* (kcal/mol)	△*G* (kcal/mol)	P_i%
**1*-a**	−2566110.008	0	35.5
**1*-b**	−2566109.930	0.078	31.0
**1*-c**	−2566109.597	0.410	17.6
**1*-d**	−2566109.535	0.473	15.9

[a] which related to the most stable conformer; [b] Boltzmann weighting factor (P_i%) based on Δ *G*.
